# Generalizability of proteomic risk prediction across biobanks reveals dependence on phenotype definitions

**DOI:** 10.64898/2026.10.01.26364038

**Published:** 2026-10-02

**Authors:** Sophie J. Parsa, Yon Ho Jee, M. Austin Argentieri, Jeremy Guez, Wenhan Lu, Evin M. Padhi, Elizabeth Kiernan, Reza Jabal, Mitja I. Kurki, FinnGen, Benjamin M. Neale, Lee Lichtenstein, Michael E. Talkowski, Stephen B. Montgomery, Niall J. Lennon, Alicia R. Martin, Mark J. Daly, Konrad J. Karczewski

## Abstract

Advances in high-throughput proteomics technologies have enabled the assessment of dynamic health states across biobank-scale cohorts. Disease prediction models built on these data have higher accuracy than baseline clinical models for a broad range of diseases, and provide avenues to understand disease pathogenesis. However, the generalizability of these prediction models across cohorts has yet to be established at scale. Here, we train models for 15 diseases in the UK Biobank (UKB; n=53,026) based on Olink proteomics data, and find high accuracy for disease prediction (mean AUC=0.74; range 0.56-0.89). These models have improved accuracy over models using only clinical factors (mean ΔAUC = 0.03), and do not depend on model architecture, with simpler models (e.g. L2) performing as well as complex models (e.g. transformer). We assess generalizability of the UKB-trained models in two external cohorts: FinnGen (FG; n=5,865) and *All of Us* (AoU; n=7,405), spanning two Olink platforms. We find that proteomics-based prevalent (classification) and incident (prediction over next 5 years) disease models largely generalize across cohorts, but performance varies across diseases. Specifically, 10 of 14 prevalent and 13 of 15 incident disease models show no significant decrease in performance across any biobank. Adjusting for demographic, ancestry, and technical covariates, we demonstrate that differences in phenotyping quality are likely the major drivers of variability across cohorts. These results indicate that proteomic risk models can be powerful and generalizable predictors of disease across multiple cohorts.

## Full Text Availability

The license terms selected by the author(s) for this preprint version do not permit archiving in PMC. The full text is available from the preprint server.

